# The Absence of CYP3A5*3 Is a Protective Factor to Anticonvulsants Hypersensitivity Reactions: A Case-Control Study in Brazilian Subjects

**DOI:** 10.1371/journal.pone.0136141

**Published:** 2015-08-20

**Authors:** Luciana Kase Tanno, Daniel Shikanai Kerr, Bernardo dos Santos, Leda Leme Talib, Célia Yamaguti, Helcio Rodrigues, Wagner Farid Gattaz, Jorge Kalil

**Affiliations:** 1 Clinical Immunology and Allergy Division, University of São Paulo, São Paulo, Brazil; 2 Laboratory of Neuroscience - LIM-27 Institute of Psychiatry, University of São Paulo, São Paulo, Brazil; 3 Laboratory of Immunology - LIM-19 Clinical Immunology and Allergy Division, University of São Paulo, São Paulo, Brazil; 4 Nursing School of the University of São Paulo, São Paulo, Brazil; 5 Center for Interdisciplinary Research on Applied Neurosciences (NAPNA), University of São Paulo, São Paulo, Brazil; Universite de Montreal, CANADA

## Abstract

Although aromatic anticonvulsants are usually well tolerated, they can cause cutaneous adverse drug reactions in up to 10% of patients. The clinical manifestations of the antiepileptics-induced hypersensitivity reactions (AHR) vary from mild skin rashes to severe cutaneous drug adverse reactions which are related to high mortality and significant morbidity. Genetic polymorphisms in cytochrome P450 genes are associated with altered enzymatic activity and may contribute to the risk of AHR. Here we present a case-control study in which we genotyped SNPs of CYP2C19, 2C9 and 3A5 of 55 individuals with varying severities of AHR, 83 tolerant, and 366 healthy control subjects from São Paulo, Brazil. Clinical characterization was based on standardized scoring systems and drug patch test. All *in vivo* investigation followed the ENDA (European Network of Drug Allergy) recommendations. Genotype was determined by real time PCR using peripheral blood DNA as a template. Of all 504 subjects, 65% were females, 45% self-identified as Afro-American, 38% as Caucasian and 17% as having non-African mixed ascendancy. Amongst 55 subjects with AHR, 44 had severe cutaneous drug adverse reactions. Of the 46 drug patch tests performed, 29 (63%) were positive. We found a strong association between the absence of CYP3A5*3 and tolerant subjects when compared to AHR (*p = 0*.*0002*, *OR = 5*.*28 [CI95% 2*.*09–14*.*84]*). None of our groups presented positive association with CYP2C19 and 2C9 polymorphisms, however, both SNPs contributed to separation of cases and tolerants in a Classification and Regression Tree. Our findings indicate that drug metabolism genes can contribute in the tolerability of antiepileptics. CYP3A5*3 is the most prevalent CYP3A5 allele associated with reduced enzymatic function. The current study provides evidence that normal CYP3A5 activity might be a protective factor to aromatic antiepileptics-induced hypersensitivity reactions in Brazilian subjects.

## Introduction

Aromatic anticonvulsants (ARA) are important drugs effective in the treatment of epilepsy, trigeminal neuralgia, and bipolar disorder [1,2]. Although well tolerated by the majority, they can cause cutaneous adverse drug reactions (ADRs) in up to 10% of patients [1]. The clinical manifestations of the antiepileptics-induced hypersensitivity reactions (AHR) vary from mild skin rashes to severe cutaneous drug adverse reactions (SCARs), such as Stevens—Johnson syndrome (SJS), toxic epidermal necrolysis (TEN) and drug reaction with eosinophilia and systemic symptoms (DRESS) [3]. These SCARs are related to high mortality and significant morbidity. The most frequently associated ARA are phenobarbital (PB), phenytoin (PHT), and carbamazepine (CBZ) [4].

The mechanisms of aromatic anticonvulsant-induced SCARs are not fully understood, but evidences suggest an immune-mediated etiology. The discovery of drug-specific T-cells in hypersensitive individuals is consistent with this hypothesis [5]. Drug patch tests (PTs) can reproduce delayed T-cell mediated hypersensitivity to drugs and are useful on determining the offending drug [5,6]. Family and twins studies suggest a genetic predisposition to these reactions [7]. Metabolism of ARA to reactive metabolites is thought to be involved in the pathogenesis of SCARs [8]. The detoxification of ARA occurs mostly in the liver where they are converted in more soluble end products which can usually be secreted by the kidneys. Cytochrome P450 enzymes, such as CYP2C19, CYP2C9 and CYP3A5, are the main enzymes in the hydroxylation of ARA and are expressed mainly in the liver as well as extra-hepatic tissues including the skin [9–13] However, in these metabolic pathways, some reactive intermediate metabolites, such as arene-oxides, can be formed [14,15]. Several Single Nucleotide Polymorphisms (SNPs) have major consequences in the expression and activity of these enzymes, however the results are still not comparable in different populations worldwide [16–18]. These informations are particularly relevant for pharmacogenetics strategies, in which the knowledge of CYP genotype may lead to individualized drug dosing and improved therapeutics.

The association between the CYP2C19, CYP2C9 and CYP3A5 allele frequencies and aromatic anticonvulsant—induced SCARs has not been studied in Brazilian subjects yet. The CYP2C19 and CYP2C9 variants have been described as being risk factors for the development of SCARs in Thai and Japanese populations [16,17]. The CYP3A5*3/*3 genotype was associated with increased half-life of CBZ in African-Americans, but no significant association was observed in Caucasians [18,19]. In addition, recent studies have demonstrated the utility of PTs as a method for the identification of both SJS/TEN and DRESS induced by ARA [20,21]. Therefore, the major goal of this study was to analyze the association between aromatic anticonvulsant—induced hypersensitivity reactions and polymorphisms of CYP2C19, CYP2C9 and CYP3A5 in Brazilian subjects and explore the clinical characteristics and results of PTs of patients with SCARs due to PB, PHT and CBZ.

## Materials and Methods

### Ethics statement

The study was reviewed and approved by the ethic local committee. All participating subjects were informed about the contents and the aims of the study and gave their written consent. After written informed consent signed, genomic DNA was extracted from peripheral blood, according to the established protocols.

### Subjects

All the 504 subjects were recruited from the University of São Paulo Faculty of Medicine Clinics Hospital, being 55 cases, 83 tolerants, and 366 healthy control subjects. Case subjects were patients with a diagnosis of hypersensitivity reactions due to PB, PHT and CBZ within 1–8 week after the drug intake, including SJS, TEN and DRESS. We excluded subjects from the same family or with history of consanguinity. All the subjects were from São Paulo, Brazil, and the ethnicity of each participant was inferred from self-reported data about their four grandparents ancestry utilizing Brazilian Institute of Geography and Statistics—IBGE—classification (White, Black, Brown, Indian and Yellow). Accordingly, we classified each individual by his/her ancestry in African-American (at least one grandparent identified as Black), Caucasian or others (non-African descendant mixed population).

### Case subjects

The DRESS group consisted of 32 patients and SJS group of 12, defined by the RegiSCAR [22–25]. We recruited 11 cases of maculopapular exanthema (MPE), defined as rash without systemic symptoms, which required the discontinuation of CBZ within 3 months after the initiation of drug therapy [23].

### Control subjects

Tolerants group consisted of 83 subjects taking ARA for at least 6 months with no clinical or biochemical signs of hypersensitivity. All the subjects were from São Paulo, Brazil.

We included samples from 366 of healthy volunteers. We did not screen either set of population controls for carbamazepine-related adverse drug reactions.

### Drug Patch Test

The PTs were performed 6 weeks to 6 months after complete healing of the adverse drug reaction, and at least 1 month after discontinuation of systemic corticosteroids. PTs were applied on the upper back, using Finn Chambers with culprit ARA. The concentrations, readings and interpretation were performed according to the European Network of Drug Allergy recommendations [26,27]. The test was also performed in 10 control subjects, all resulted negative. The medications or conditions that could affect the interpretation of the test followed the guidelines of the International Contact Dermatitis Group.

### Genotyping

DNA was extracted from peripheral blood by salting-out [28]. SNPs characterizing CYP2C19*2, CYP2C19*3, CYP2C19*17, CYP2C9*2, CYP2C9*3 and CYP3A5*3 alleles (Table 1) were genotyped using TaqMan allele-specific polymerase chain reactions (Life Technologies). Amplification reactions were as follow: TaqMan PCR Mastermix 1x/μL, TaqMan SNP genotyping assay 1x/μL, genomic DNA 1ng/μL, ultrapure water to complete 7μL volume. rs12248560 required 500mM of Betaine (Sigma Aldrich) in the final reaction.

**Table 1:** Candidate CYP genes, alleles, polymorphisms identification and enzymatic activity.

**CYP2C19**		
Allele	Ref SNP ID	Enzymatic activity
*2	rs4244285	Null
*3	rs4986893	Null
*17	rs12248560	Increased
*17	rs11188072	Increased
**CYP2C9**		
*2	rs1057910	Decreased
*3	rs1799853	Decreased
**CYP3A5**		
*3	rs776746	Decreased

Allele discrimination was evaluated in a Line Gene 9600 (BIOER Technology CO.) comparing amplification curves and fluorescence levels before and after amplification (45 cycles of 15 seconds at 95°C and 1min at 60°C).

The candidate CYP genes were chosen according to the described association to the aromatic anticonvulsant-reactions [14,15,18,19,29] in different populations and the importance in the metabolism of these drugs (Table 1).

According to the alleles presented, each subject was classified with normal (EM), decrease (IM/PM) or increased (UM) function for each enzyme (Table 2).

**Table 2:** Candidate CYP genes, corresponding predicted phenotype and enzymatic activity according to the allele combination (EM = normal metabolizers, IM and PM = decrease function, UM and UMH = increase function).

Gene	Predicted Phenotype	Enzymatic activity of allele combination
**CYP2C19**	EM	Normal+Normal; Increased+Decreased
	IM	Normal+Decreased
	UM	Increased+Increased
	UM heterozygote	Increased+Normal
	PM	Decreased+Decreased
**CYP2C9**	EM	Normal+Normal
	IM	Normal+Decreased
	PM	Decreased+Decreased
**CYP3A5**	EM	Normal+Normal
	IM	Normal+Decreased
	PM	Decreased+Decreased

### Statistical Analysis

The data is available as a supporting information table (S1 Table). Descriptive statistical methods (mean, median, and frequency) were applied to analyze the demographic data. Analyses of the difference in frequencies across groups (sex and clinical manifestation) were performed with the chi-squared test or analysis of variance. CYP phenotypes were compared between diagnostic groups (Healthy, Tolerant and Cases) with Pearson's Chi-squared test. Significance was set at p<0.05. The CYP phenotypes were also entered in a Classification and Regression Tree (CART) model to predict tolerant cases. Variable selection was based on Gini impurity index and pruning was conducted based on 10-fold cross-validation to avoid over fitting. All analyses were conducted with the aid of the R software version 3.2.0.

## Results

### Clinical characteristics of aromatic anticonvulsant—induced hypersensitivity reactions patients and control subjects

There were no significant differences in the demographic data between groups as shown in Table 3.

**Table 3:** Demographic data of cases and controls subjects.

Variables	Cases (N = 55)	Tolerants (N = 85)	Healthy Controls (N = 366)	Total (506)
Categorical Variables	N (%)	N (%)	N (%)	N (%)
**Gender**				
Female	42 (76)	47 (55)	293 (80)	382 (75)
Male	13 (24)	38 (45)	73 (20)	124 (25)
**Age**				
Young adults (18–40 years)	23 (42)	26 (30)	147 (40)	196 (39)
Adults (40–60 years)	25 (46)	44 (52)	179 (49)	248 (49)
Elderly(> 61 years)	7 (12)	15 (18)	40 (11)	62 (12)
**Ethnicity**				
Caucasian	14 (26)	20 (24)	161 (44)	195 (38)
Afro-American	29 (53)	41 (48)	154 (42)	224 (45)
Others	12 (21)	24 (28)	51 (14)	87 (17)

The severities of cutaneous manifestation of the reactions varied from MPE to exfoliative dermatitis. As shown in Table 4, 32 patients were validated as DRESS, 12 as SJS and 11 as MPE. CBZ was involved in 82% of the reactions, both mild and SCARs. The late onset of the reaction was important in cases of DRESS, but happened in low proportion in SJS. No MPE happened after 4 weeks of drug intake. SCARs showed to be systemic reactions with internal organ involvement requiring long-term hospitalization, mainly in DRESS cases but also in SJS (Table 4). Most of the cases, both mild and severe, were treated with systemic corticosteroids and anti-histamines. Intravenous immunoglobulin (IgIV) was exclusively indicated to SJS cases. We found no association between ethnicity and clinical status (p = 0.231).

**Table 4:** Clinical patterns of aromatic anticonvulsant-induced reactions and demographic characteristics (DRESS = Drug Reaction with eosinophilia and systemic symptoms, SJS = Stevens-Johnson Syndrome, MPE = Maculopapular exanthema, IgIV = Intravenous immunoglobulin).

	DRESS (N:32)	SJS (N:12)	MPE (N:11)	TOTAL (N:55)
**Mean age**	49.7	48.3	47.7	43.6
**Sex** (M/F)	26/6	7/5	9/2	42/13
**Onset of the reaction**				
< 4 weeks (%)	13 (40)	11 (92)	11 (100)	35 (64)
> 4 weeks (%)	19 (60)	1 (8)	0 (0)	20 (36)
**Drug involved**				
Phenobarbital	4 (13)	2 (17)	1 (9)	7 (13)
Phenytoin	2 (6)	1 (8)	0 (0)	3 (5)
Carbamazepine	26 (81)	9 (75)	10 (91)	45 (82)
**Clinical manifestations**				
Cutaneous (%)	32 (100)	12 (100)	11 (100)	55 (100)
Gastrointestinal (%)	32 (100)	6 (50)	0 (0)	38 (69)
Respiratory (%)	5 (15)	4 (34)	0 (0)	9 (16)
Fever (%)	32 (100)	11 (91)	2 (18)	45 (82)
**Mean time of hospitalization** (days)	20	12	0	24
**Treatment**				
Anti-histamines (%)	32 (100)	8 (67)	11 (100)	51 (93)
Systemic corticosteroids (%)	32 (100)	12 (100)	8 (72)	52 (95)
IgIV (%)	0 (0)	5 (41)	0 (0)	5 (9)
**Drug patch test** (N: 46)				
Positive	18 (72)	7 (58)	4 (45)	29 (63)
Negative	7 (28)	5 (42)	5 (55)	17 (37)
Not Done	7	0	2	9 (16)

Of all 46 PTs, 29 (63%) were positive and 63% of positive results were in DRESS. The PTs was not performed in 9 cases due to the difficulties on the clinical follow-up or if the patient had contraindications to the procedure. Apparently grade of positivity of PTs did not correlated with the intensity of reaction nor the internal organ involvement, however, positive reactions occurred in the more severe cases. There was no association between positivity of the tests and skin color or reported ethnicity. None of the patients presented adverse reactions during the in vivo procedure.

### CYP3A5, CYP2C9 and CYP2C19 genotyping and aromatic anticonvulsant—induced hypersensitivity reactions

On the basis of our hypothesis that the polymorphisms of CYP2C19, CYP2C9 and CYP3A5 are associated with aromatic anticonvulsant—induced hypersensitivity reactions, we genotyped the CYP2C19*2, CYP2C19*3, CYP2C19*17, CYP2C9*2, CYP2C9*3 and CYP3A5*3 alleles in all subjects included in the study (Tables 1 and 2). The allele frequencies and its distribution among the enzymatic phenotype and the studied groups are shown in Table 5.

**Table 5:** Phenotipic frequencies for cytochrome P450 (CYP) 2C9, 2C19 and 3A5 in healthy controls, cases and tolerants (EM = normal metabolizers, IM and PM = decrease function, UM and UMH = increase function) as predicted by SNP genotype.

Gene (SNP)	Enzymatic Phenotype Group	Phenotypic Frequency (%)			P Value
		Healthy controls (%)	Cases (%)	Tolerants (%)	
**CYP2C9** (rs1057910, rs1799853)	EM	251 (71.1)	43 (81.1)	54 (65.9)	
	IM	97 (27.5)	8 (15.1)	25 (30.5)	0.083
	PM	5 (1.4)	2 (3.8)	3 (3.7)	
**CYP2C19** (rs4244285, rs4986893, rs12248560, rs11188072)	EM	163 (48.4)	24 (47.1)	41 (51.3)	
	IM/PM	79 (23.4)	12 (23.5)	20 (25)	0.945
	UM/UMH	95 (28.2)	15 (29.4)	19 (23.8)	
**CYP3A5** (rs776746)	EM	37 (10.6)	8 (14.8)	38 (46.3)	
	IM	122 (34.9)	23 (42.6)	19 (23.2)	**0.0005** OR = 4.9
	PM	191 (54.6)	23 (42.6)	25 (30.5)	

**Fig 1:**
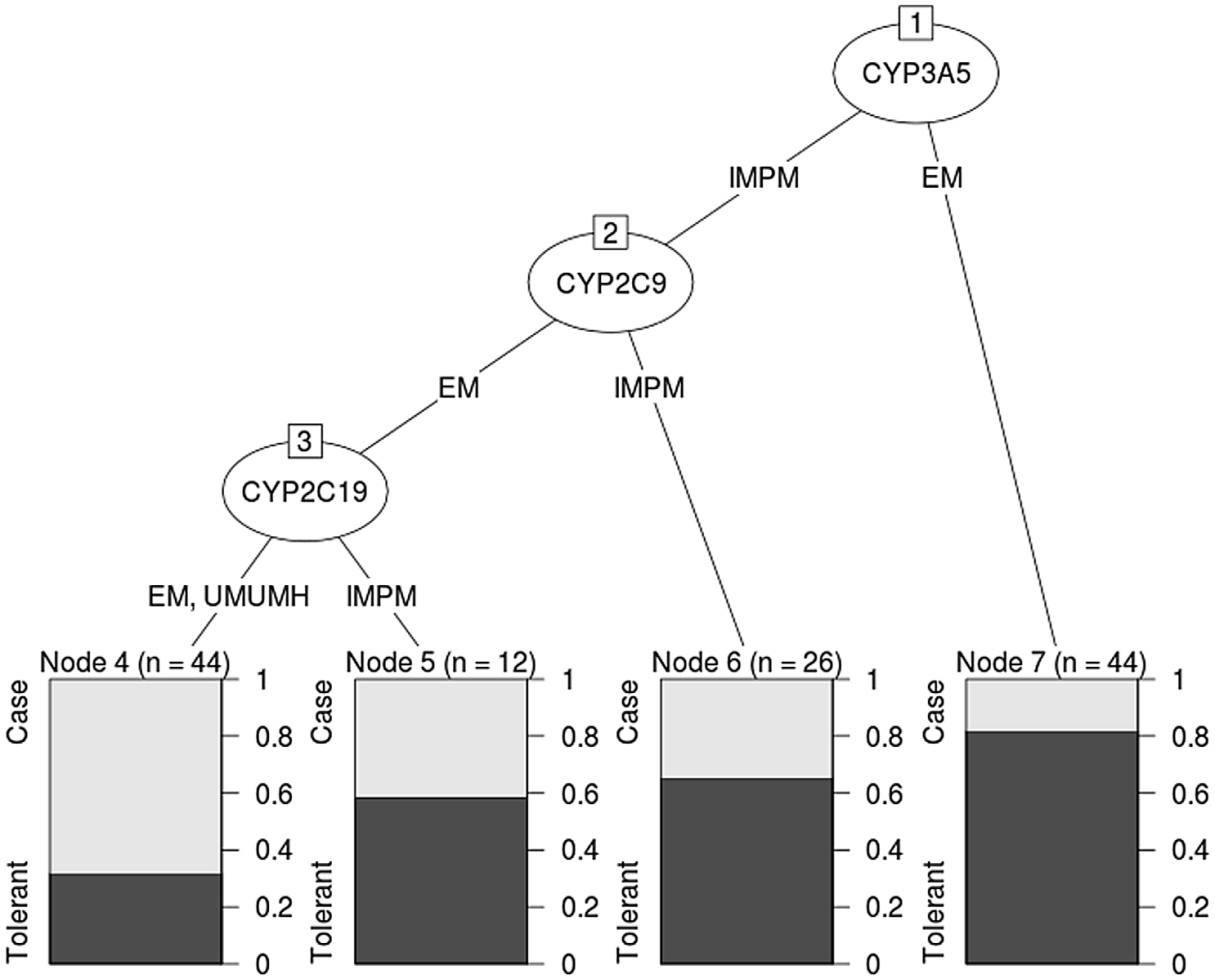
Classification and regression tree: CYP3A5 phenotype discriminates between cases and tolerants (EM = normal metabolizers, IM and PM = decrease function, UM and UMH = increase function).

We showed a strong association between the absence of CYP3A5*3 and tolerant subjects when compared to cases (p = 0.0002, OR = 5.28 [CI95% 2.09–14.84]). However, we observed homogeneous distribution of variants of CYP2C19 and 2C9 in our case and control groups (Table 5). The classification and regression tree showed that non-decreased function of CYP3A5 (absence of *3 allele; EM), was the most important factor to classify tolerant individuals (Fig 1). On the basis of this result, the absence of CYP3A5*3 has a putative sensitivity of 81.1% as a protective factor to aromatic antiepileptics-hypersensitivity reactions, whereas the presence of IM or PM phenotypes is split in 54.32% cases and 45.67% tolerants.

Given the fact that CYP2C19, CYP2C9 and CYP3A5 enzymes are expressed in the skin, we analyzed the association between genotype and the results of PT. Even without statistically significant associations, we observed a predominance of low function phenotype of CYP3A5, normal function of CYP2C9, and increased function of CYP2C19 in positive results.

## Discussion

This study is the first and most broad case-control study ever conducted towards the association of CYP alleles and aromatic antiepileptics-induced hypersensitivity reactions in Brazilian subjects. Our data add to the growing evidence of different CYP alleles role in immune-mediated ADRs, such as drug-induced hypersensitivity [5,6]. Genetic variation in genes involved in their metabolism may result in the increase of arene oxide, which potentially initiates immune responses [8]. On the other hand, the immune response to CBZ in AHR has been associated with the drug itself and not its metabolites [6].

Genetic polymorphisms of CYP2C19 are highly present in Asian population and the most common variant resulting in poor metabolism are CYP2C19*2 and CYP19*3 [16,30]. The allele frequency of CYP2C9*2 and CYP2C9*3 vary among different populations, but seems to be more frequent in Caucasians. The CYP3A5 gene is highly polymorphic and the allele CYP3A5*3 is the most prevalent related to the loss of activity. The prevalence of this allele appears to be higher in Caucasians when compared to African Americans. The CYP3A5*3/*3 genotype was associated with increased half-life of CBZ in African-Americans, but no significant association was observed in Caucasians [16,29,31].

We found that the absence of the CYP3A5*3 is an important protective factor of the full spectrum of aromatic antiepileptics-induced hypersensitivity reactions in Brazilian subjects. The CYP3A5*3 has been shown to be related to the decreased function of this enzyme. The association of drug tolerance with normal activity of CYP3A5 supports previous works indicating that immune response to CBZ in AHR is due to the drug itself and not its metabolytes [6,32,33]. Whereas we demonstrated that the absence of this allele is related with increased tolerability of these drugs, the similar proportion of CYP3A5*3 in both cases and controls with IM/PM phenotypes underlined that it cannot be considered an isolated risk factor for the development of antiepileptic hypersensitivity reactions.

However, the presence of the CYP3A5*3 allele is neither necessary nor sufficient for the development of these reactions. Although CYP3A5*3 is the most prevalent CYP3A5 allele with reduced function, it is not the only one [19]. Thus, it remains to be evaluated if cases in our sample that were considered with normal CYP3A5 function (EM) are actually carriers of rarer non-functional alleles. Most studies published in the last years, which have been searching for genetic risk factors for the development of drug-induced hypersensitivity reactions, showed the predictive value of HLA alleles [34,35]. The current study now provides evidence suggesting that the absence of the CYP3A5*3 is a protective factor to aromatic antiepileptics-induced hypersensitivity reactions in Brazilian subjects. The worldwide distribution of CYP3A5*3 provides a remarkable example of population diversity, with allele frequencies ranging from 0.14 among sub-Saharan Africans to 0.95 in European populations [18]. The Brazilian population, in excess of 195 million people, has major ancestral roots in Europe, Africa and America, nevertheless, we didn’t found significant association between ethnicity and clinical status. It has been recently reported that the genetic background of the Brazilian population is less heterogeneous than previously believed, with a high percentage (60–77%) of caucasian background [36]. This study is the first to show the association between CYP3C5*3 and aromatic anticonvulsant—induced reactions in subjects from São Paulo, Brazil. CYP3C5*3 may have a potential role in future screening before prescribing aromatic anticonvulsant, especially CBZ. Since we do not know the responsiveness of the healthy population to anticonvulsants we calculated negative (NPV) and positive predictive value (PPV) for the absence of CYP3A5*3 to predict tolerability considering cases and tolerants subjects. In this scenario it shows a NPV of 51% and PPV of 82%. According to our data, testing would prevent 4 cases of adverse reaction in every 5 prescriptions of anticonvulsants (NNT = 4 [2.1–5.5]).

We did not found association between CYP2C19 and CYP2C9 variants and cases in our study. The CYP2C9 and CYP2C19 variants have been described as risk factors for the development of SCARs in Thai and Japanese populations [16,17]. In addition, CYP2C9 has been shown to be associated with PHT metabolism and elimination, but not with PB or CBZ and CYP2C19 may be the major metabolic pathway of PB [15]. Most cases of this study were SCARs due to CBZ in a Brazilian cohort of mixed ethnic background, which might explain the discrepancies with the literature. Patch Tests are the only in vivo diagnostic method available to SCARs up to now, with positive reactions in 60–100% of the cases studied [20,21]. Nevertheless, the reactivity depends on the type of the cutaneous adverse drug reactions. The positivity of PTs in our study varied according to the clinical pattern, being more positive (72%) in cases of DRESS. The CYP2C9, CY2C19 and CYP3A5 enzymes have been described as being expressed in human skin [37]. Here, we hypothesized that they can influence in the positivity of the ARA patch test, however we found no association between functional CYP3A5 genotype in individuals with positive PTs. CYP activation is a possible mechanism for allergic contact dermatitis (ACT), a complex syndrome representing immunological responses to cutaneous and/or systemic exposure to protein-reactive chemicals. Recently, many drug-induced hypersensitivity reactions such as DRESS and SJS have been classified in a similar pathophysiologic scenario as the ACT.

We are aware that the current findings may not be taken as isolated genetic factors to determine lower risk on developing AHR. Considering the ongoing knowledge on the specific HLA alleles as risk factors for SCARs, we would expect that combined analysis of CYP and HLA data could help identify subjects with higher or lower risk of developing AHR. HLA-A*3101 has been associated with AHR in European population while HLA-B*1502 in Chinese. Given the relative rarity of those two alleles in the Brazilian population [38] and the ethnic specificity they present we do not believe that they would substantially modify the present analysis. However, further studies are necessary in order to rule out this possibility.

Some limitations of the present study are similar to the majority of the association studies linking genetic factors and antiepileptics-induced cutaneous adverse reactions, such as the limited number of cases, the population included were exclusively of subjects living in São Paulo and the ancestry was based on self-identification. Due to the self-identified admixed ancestry in our cohort, we believe that the current data may require replication by future multicentric multinational collaborations with accurate phenotypic characterization studies in populations of other ancestry subjects.

We therefore suggest that consideration be given to the absence of CY3A5*3 as a genetic preventive marker of aromatic anticonvulsants hypersensitivity reactions.

## Supporting Information

S1 Table Data utilized in this study in a CSV format file. Ethnicity (AA—afro american, C—caucasian, O—others), Clinical_Pattern (0 –Healthy, 1 –Case, 2 –Tolerant), Clinical_Pattern_Severity (0 –Healthy, 1 –Severe Reaction, 2 –Tolerant, 3 –Mild reaction), Type_Of_Reaction (0 –Healthy, 2 –Tolerant, 3 –Mild reaction, 4 –DRESS, 5 –Steven Johnson), Contact (P—Positive, N—Negative, ND—Not Done).
(CSV)
