# Correction: The Absence of CYP3A5*3 Is a Protective Factor to Anticonvulsants Hypersensitivity Reactions: A Case-Control Study in Brazilian Subjects

**DOI:** 10.1371/journal.pone.0139861

**Published:** 2015-09-29

**Authors:** Luciana Kase Tanno, Daniel Shikanai Kerr, Bernardo dos Santos, Leda Leme Talib, Célia Yamaguti, Helcio Rodrigues, Wagner Farid Gattaz, Jorge Kalil

The following information is missing from the Funding section: Fundação de Amparo à Pesquisa do Estado de São Paulo also provided support in the form of grant 2013/01352-8.

The complete, correct funding information is as follows: This work received funding from Fundação de Amparo à Pesquisa do Estado de São Paulo (FAPESP- http://fapesp.br/) under the grant numbers 2011/22748-1 and 2013/01352-8. The funders had no role in study design, data collection and analysis, decision to publish, or preparation of the manuscript.
